# An Account of the Most Important Diseases Peculiar to Women

**Published:** 1849-02

**Authors:** 


ART. XI.—*An Account of the most important Diseases peculiar to Women.* By ROBERT GOOCHE, M. D., with Illustrations. Philadelphia: Ed. Barrington and Geo. D. Haswell: 1848. 8vo. p. 322.

This is the second edition published in this country of a work which was exceedingly well received when it first appeared. It is issued without note or comment, but we feel quite sure that it needs no recommendation from editors or professed critics to give it currency with the profession. Its author was a man of vigorous and independent mind, who saw correctly, and wrote with clearness and spirit of subjects which he thoroughly understood. The present edition is got up in good style, the paper being excellent, and the type large and legible. We believe our readers will be glad to learn that this work is once more within their reach.

